# Mitigating Salinity Stress in Quinoa (*Chenopodium quinoa* Willd.) with Biochar and Superabsorber Polymer Amendments

**DOI:** 10.3390/plants13010092

**Published:** 2023-12-27

**Authors:** Imed Derbali, Walid Derbali, Jihed Gharred, Arafet Manaa, Inès Slama, Hans-Werner Koyro

**Affiliations:** 1 Institute of Plant Ecology, Justus Liebig University Giessen, 35392 Giessen, Germany; imed.derbali@bio.uni-giessen.de (I.D.); derbaliwalda@gmail.com (W.D.); gharred.jihed@bio.uni-giessen.de (J.G.); 2 Laboratory of Extremophile Plants, Center of Biotechnology of Borj Cedria, Hammam-Lif 2084, Tunisia; manaaarafet@gmail.com (A.M.); slama_ines@hotmail.fr (I.S.); 3 Faculty of Mathematical, Physical and Natural Sciences of Tunis, University of Tunis El-Manar, Tunis 1068, Tunisia

**Keywords:** saline lands, SAP, BC, photosynthesis, CO_2_/H_2_O gas exchange, chlorophyll fluorescence, antioxidant enzyme activity, reactive oxygen species, oxidative stress, ascorbate, proline, MDA

## Abstract

In agriculture, soil amendments are applied to improve soil quality by increasing the water retention capacity and regulating the pH and ion exchange. Our study was carried out to investigate the impact of a commercial biochar (Bc) and a superabsorbent polymer (SAP) on the physiological and biochemical processes and the growth performance of *Chenopodium quinoa* (variety ICBA-5) when exposed to high salinity. Plants were grown for 25 days under controlled greenhouse conditions in pots filled with a soil mixture with or without 3% Bc or 0.2% SAP by volume before the initiation of 27 days of growth in hypersaline conditions, following the addition of 300 mM NaCl. Without the Bc or soil amendments, multiple negative effects of hypersalinity were detected on photosynthetic CO_2_ assimilation (Anet minus 70%) and on the production of fresh matter from the whole plant, leaves, stems and roots (respectively, 55, 46, 64 and 66%). Moreover, increased generation of reactive oxygen species (ROS) was indicated by higher levels of MDA (plus 142%), antioxidant activities and high proline levels (plus 311%). In the pots treated with 300 mM NaCl, the amendments Bc or SAP improved the plant growth parameters, including fresh matter production (by 10 and 17%), an increased chlorophyll content by 9 and 13% and A_net_ in plants (by 98 and 115%). Both amendments (Bc and SAP) resulted in significant salinity mitigation effects, decreasing proline and malondialdehyde (MDA) levels whilst increasing both the activity of enzymatic antioxidants and non-enzymatic antioxidants that reduce the levels of ROS. This study confirms how soil amendments can help to improve plant performance and expand the productive range into saline areas.

## 1. Introduction

Soil salinization is a major environmental challenge that is threatening agriculture across the world. The demands on crop yield have risen sharply worldwide to keep up with the rapidly expanding human population over the past twenty years [1,2]. Rising salinity threatens food security, access to drinking water and coastal biodiversity [3]. More than 424 million hectares of topsoil (0–30 cm) and 833 million hectares of subsoil (30–100 cm) from 118 countries covering 85% of the global land area are salt-affected, and these are mainly located in arid and semi-arid climate zones [4]. Worldwide, currently approximately 600 million people live in low-lying coastal areas that will be strongly affected by progressive salinization [5,6]. In addition, the global increase in temperature caused by greenhouse gases is leading, especially in arid and semi-arid countries, to enhanced evapotranspiration and decreased precipitation, resulting in a decrease in salt-leaching capacity [3,7,8,9]. It can be predicted that the salinization of agricultural land will continue to rise due to climate change and poor irrigation practices [10,11], with negative impacts on crop productivity [12].

A soil is classified to be saline when the electric conductivity (EC) of the soil solution is above 4 dS m^−1^ (equivalent to 40 mM NaCl), which creates an osmotic potential around −0.2 MPa and significantly decreases the yields of most crops [4,13]. There is an urgent need to combine salt-tolerant crop species (genetic selection) with appropriate agronomic methods by studying the physiological mechanisms and interactions within the soil–plant–atmosphere continuum (SPAC) [14]. 

One major adverse effect of high salinity is to decrease the uptake of potassium, magnesium, calcium, phosphorus and nitrate, and this leads to ion cytotoxicity, an imbalance of nutrients and osmotic stress [15,16]. These can affect the rate of cell expansion in growing tissues, seed germination, growth and development, flowering and fruiting [17,18]. It can also lead to injury of photosynthetically active leaves by causing chlorosis and triggering leaf senescence and a decline in productivity [19]. Photosynthetic rates may also decrease through stomatal closures for maintenance of a positive water balance, through ion-specific effects on the photosynthetic apparatus or the overproduction of reactive oxygen species [20,21]. 

Plant salt-resistance mechanisms can be grouped into cellular homeostasis, stress damage control and growth regulation [22]. Depending on the ability of plants to grow in saline environments, they are classified as either salt-sensitive or salt-resistant. Salt-resistant plants need mechanisms to avoid ionic toxicity, metabolic imbalances and nutritional limitations, to adapt to a hyperosmotic environment without overheating and to control the genesis of reactive oxygen species (ROS). Indeed, the photosynthetic apparatus is an important site for the production of ROS-free radicals, including superoxide (O_2_^●−^), hydrogen peroxide (H_2_O_2_) and hydroxyl (HO^●^) [23,24]. The presence of enzymatic and non-enzymatic antioxidants forms the first line of defense against the reactive oxygen species (ROS) generated by abiotic stress (salinity, drought, temperature) [25]. It includes enzymatic antioxidants such as superoxide dismutase (SOD), peroxidase (POX), catalase (CAT), glutathione peroxidase (GPX) and ascorbate peroxidase (APX), which interact in a sequence to ameliorate scavenging of the reactive oxygen species [26,27]. The impact of the highly toxic ROS can be very severe on photosynthesis and cell metabolism by oxidizing the membranes of vital biomolecules [28,29,30], proteins and nucleic acids [31]. Cell membranes play a vital role in cellular transport and plant resistance [29,32]. To escape these toxic effects, salt-resistant plants have developed molecular defense strategies to ensure a certain balance between the production and trapping of these species by reducing their formation and increasing their elimination [33].

Most of our high-yielding crop varieties are salt-sensitive [34], and salt-resistant halophytic crop development has been on the agenda for decades. In this context, the facultative halophyte quinoa (*Chenopodium quinoa* Willd.) has emerged as an important model (cash) crop halophyte and is currently considered a ‘high potential’ crop suited for sustainable agriculture [35]. Over the years, the global production of quinoa increased significantly and exceeded 147 thousand metric tons in 2021. Peru and Bolivia are the leading quinoa-producing countries [36]. In 2020, the United States imported approximately 28.3 million pounds of quinoa, predominantly from the Andean region [36]). Quinoa is marked as a superfood, combining high-quality proteins, high fiber contents [37] and all nine essential amino acids, and is rich in vitamins (A, B2, and E), important minerals (Ca, Fe, Cu, Mg and Zn), isoflavones and high-quality lipids [38]. In addition, quinoa seeds offer a wide range of chemical compounds like saponins and therapeutic properties (anti-inflammatory activity), as well as a low glycemic index, and contain significant amounts of (mono- and poly-) unsaturated fatty acids (omega-3 and omega-6), known for their protective effect on the cardiovascular system [39]. 

The suitability of quinoa for the present study is a result of its capability to adapt to diverse agroecological conditions worldwide [40]. Quinoa developed various defense mechanisms to resist abiotic stress, such as drought, wind, salinity and biotic stresses, such as various diseases, parasites and pests. 

However, in order to carry out successful cultivation of quinoa in a hypersaline environment, it is reasonable and expedient to optimize the soil’s quality and, thus, the ability of this plant species to resist this harsh environment. One possibility to enhance the soil quality is an amendment with biochar (Bc) [41]. Bc can significantly increase the organic matter content and enhance the water retention capacity, nutrient uptake, soil aeration and respiration (see literature cited [41]). Bc provides, during low water supply, better conditions for the synthesis of organic solutes, prevents desiccation with improved turgidity and reduces oxidative stress by high water-use efficiency [42,43]. It has the same high salt sorption effect as charcoal [44]. It is able to mitigate the negative effects of salinity, supports the reduction of Na^+^ uptake and facilitates Na^+^ exclusion in plants. 

Another possibility to improve the quality of poor soils is with the addition of a superabsorbent polymer (SAP). SAP has been used in agriculture for decades worldwide, especially in areas with sandy soil and low rainfall. It is established in the literature that the utilization of SAP as a soil amendment can improve the soil structure, such as its water-holding capacity, plant-available water content and finally, plant performance because of its hydrophilic three-dimensional network [45]. The function of this network is to retain water as well as water-soluble fertilizers and redistribute it on demand to the plant [46,47]. It was also shown that SAP is able to retain Cl^−^ and Na^+^ in the soil solution and that the exchangeable K^+^ contained therein supports a tolerable K^+^/Na^+^ balance in salinized plants [48].

Both amendments, Bc and SAP, share a high potential to expand the area suitable for agriculture and to improve the performance and yield of quinoa in a saline environment. Currently, no data in the literature are available regarding the causality, specific impact and effectiveness of Bc and SAP on the response of *Chenopodium quinoa* Willd. to hyperosmotic salinity. We expect that both the addition of BC and SAP will enable quinoa to minimize the impact of salinity on growth and water relations, stabilize photosynthesis and buffer the development of ROS with photoprotective mechanisms. Therefore, the aim of the present study was to investigate and compare the potential of the Bc and SAP amendments to enhance the growth and yield, ion relations, chlorophyll content, coordination of light-dependent reactions of photosynthesis response, gas exchange and the ROS defense of *Chenopodium quinoa* (Willd.) grown in a hyperosmotic NaCl salinity. 

## 2. Results

### 2.1. Growth

**Figure 1:**
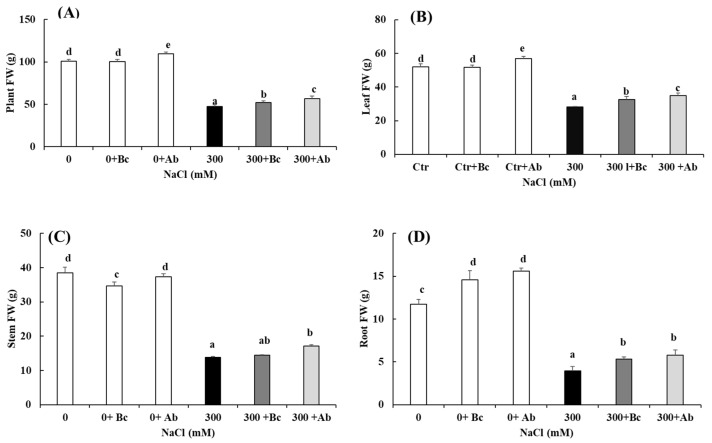
Effect of biochar or absorber amendments on plant growth parameters of *Chenopodium quinoa* (variety ICBA-5) after culture without any NaCl addition or 27 days of salt treatment. (**A**) Fresh weight of the whole plant, (**B**) leaf, (**C**) stem and (**D**) root. Values represent mean ± SE (n = 5), and the different letters a to e indicate significant differences between the treatments. Control (0), 300 mM NaCl (300), biochar (Bc), absorber (Ab).

A salinity treatment of 300 mM NaCl over 27 days significantly affected the whole plant’s fresh weight (FW), leaf FW, stem FW (except the stem FW at 0 + Bc), and the root FW decreased, respectively, by 55%, 46%, 64% and 66% as compared to the control treatment (0 mM NaCl) (Figure 1A–D). Bc and SAP had a positive impact on the plant development at 0 and 300 mM NaCl. Bc supported, at 0 mM NaCl, a higher root growth and at 300 mM NaCl, the increase of all four growth parameters. The SAP amendment led to an increase in all growth parameters of both salinity levels, with the exception of the stem FW at 0 mM NaCl. 

### 2.2. Chlorophyll Content

**Figure 2:**
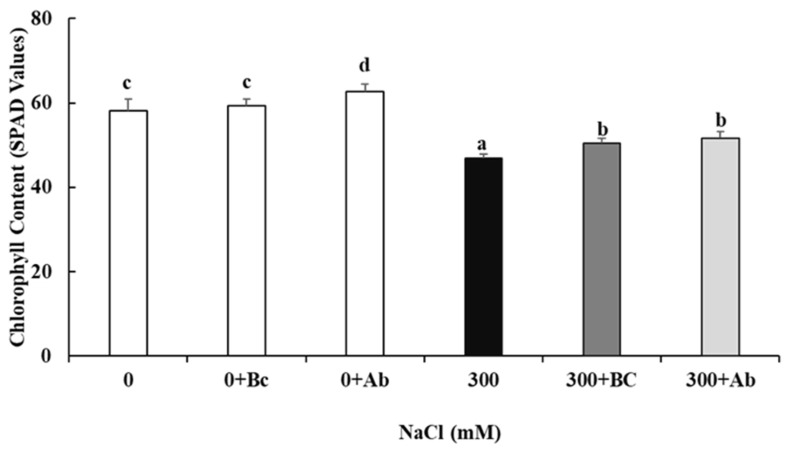
Effect of biochar or absorber amendments on chlorophyll content in *Chenopodium quinoa* Willd. (ICBA-5 variety) without any NaCl addition or 27 days of salt treatment. Values represent mean ± SE (n = 5), and the different letters a to d indicate significant differences between the treatments. Control (0), 300 mM NaCl (300), biochar (Bc), absorber (Ab).

As shown in Figure 2, 300 mM NaCl salinity led the culture to have a significant decrease in the leaf chlorophyll content. However, the amendment of the absorber significantly enhanced the chlorophyll content with the 0 NaCl treatment (0 + Ab) and at the 300 NaCl level (300 + Ab). The latter response was also observed for the Bc treatment with hyperosmotic salinity (300 + Bc). 

### 2.3. Proline and MDA

**Figure 3:**
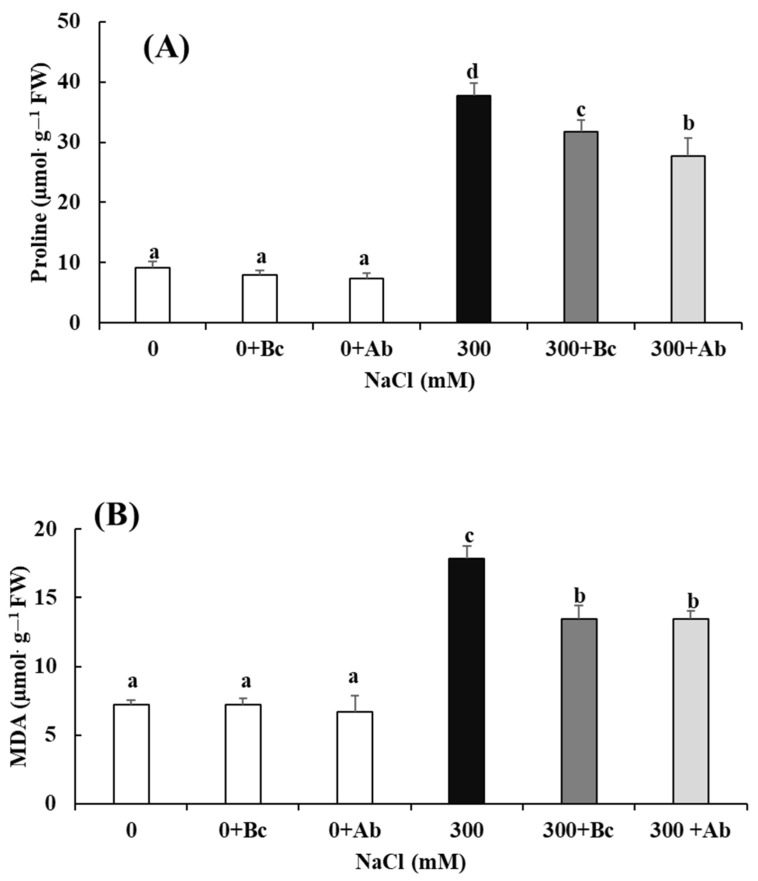
Effect of biochar or absorber amendments on proline (**A**) and MDA (**B**) contents in leaves of *Chenopodium quinoa* Willd. (ICBA-5 variety) after culture without any NaCl addition or 27 days of salt treatment. Values represent mean ± SE (n = 5), and the different letters a to d indicate significant differences between the treatments. Control (0), 300 mM NaCl (300), biochar (Bc), absorber (Ab).

In fact, the lowest proline and MDA contents were observed at 0 mM NaCl with or without the amendment. The highest proline and MDA contents were observed with the treatment of 300 mM NaCl salinity without any amendment (37.64 µmol·g^−1^ FW and 17.85 µmol·g^−1^ FW, respectively) (Figure 3A,B). Both the Bc and SAP amendments led to a significant decrease in both parameters with 300 mM NaCl salinity.

### 2.4. Gas Exchange

The plant leaves reached light saturation in the 0 mM NaCl treatment without amendment of a net CO_2_ assimilation rate (A_net_) of 21.5 ± 1.79 µmol m^−2^ s^−1^ (Table 1). Only the SAP led to a significant increase of A_net_ (26.95 ± 2.42 µmol m^−2^ s^−1^) in the 0 mM NaCl treatment. Salinity generally induced a significant inhibition of photosynthesis. Both the Bc and SAP amendments buffered the reduction and reached twice as high A_net_ values as the plants receiving the treatment without amendment.

There was a significant correlation between the values of Anet, transpiration (Tr) and stomatal conductivity (g_s_). High g_s_ led to high A_net_ and Tr. Consequently, there was no significant impact on the water-use efficiency in all treatments apart from very low values in the SAP treatment at 0 mM NaCl (3.97 + 0.41 µmol CO_2_ mmol H_2_O^−1^).

It was noticeable that the Bc amendment in the 0 NaCl treatment led to a significantly lower substomatal CO_2_ concentration (Ci) as in both other treatments at the same salinity. Consequently, only these treatments at 300 mM NaCl showed significant decreases in Ci. 

### 2.5. Quantum Yields and Energy Conversion in PSII

**Figure 4:**
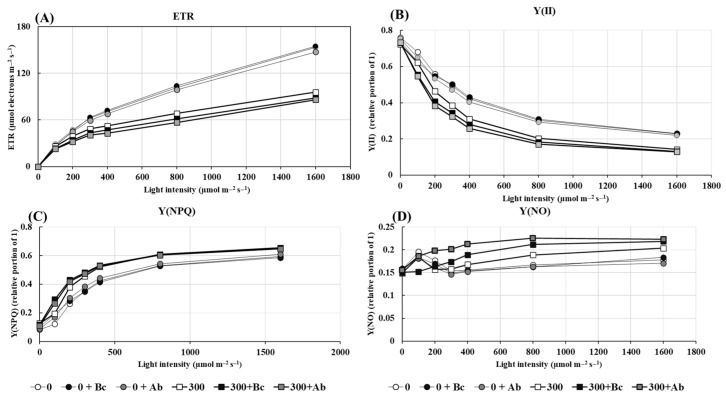
Impact of biochar or absorber amendments on the energy distribution of incident light in leaves of *Chenopodium quinoa* Willd. (ICBA-5 variety) after culture without any NaCl addition or 27 days of salt treatment. (**A**) Electron transport rate (ETR); (**B**) quantum yield of photosystem II (Y(II)); (**C**) non-photochemical quenching (Y(NPQ)) and (**D**) fraction of energy that is passively dissipated in the form of heat and fluorescence (Y(NO)). Control (0), 300 mM NaCl (300), biochar (Bc), absorber (Ab).

**Figure 5:**
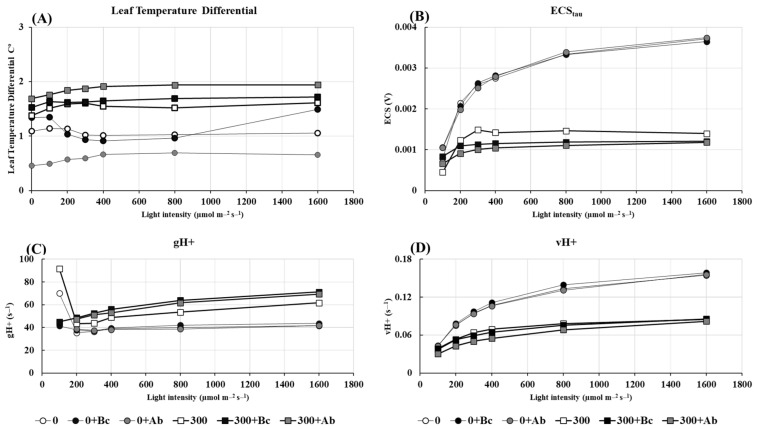
Impact of biochar or absorber amendments on leaf temperature differential (**A**), ECS_tau_ (**B**), the steady-state rate of proton flux (gH^+^) (**C**) and proton conductivity (vH^+^) (**D**) of the chloroplast ATP synthase in leaves of *Chenopodium quinoa* Willd. (variety ICBA-5) after culture without any NaCl addition or 27 days after salt treatments began. Control (0), 300 mM NaCl (300), biochar (Bc), absorber (Ab).

The salt-related decrease of A_net_ (see Table 1) was reflected by a reduction of ETR and Y(II) and an increase of Y(NPQ) and Y(NO) (Figure 4). The major response of photosynthesis to hyperosmotic salinity was a cross-culture relocation of energy from photochemical to non-photochemical use and passive dissipation. Moreover, there was a close correlation between the salt-induced increase of Y(NPQ) and the increase in the leaf temperature differential (Figure 5A). 

The amendment of Bc or SAP had no significant impact on the electron transport rate (ETR) of PSII in the 0 NaCl treatments (Figure 4A). However, both induced a slight reduction of ETR at a 300 mM NaCl salinity. This effect was accompanied by a reinforced reduction of Y(II) and an increase of Y(NO).

### 2.6. PSI Performance Activity

Salinity, despite the Bc and SAP amendments, had a strong impact on the chlorophyll fluorescence and absorbance-based parameters. The steady-state rate of proton translocation (gH^+^) through the chloroplast ATP synthase, was measured as magnitude of electrochromic shift (ECS_tau_), and regardless of the amendment, significantly elevated (up to more than 3 times) higher than the control plants, 300 mM NaCl (Figure 5C). The increase of ECS_tau_ was accompanied by a decrease in the steady-state rate of proton flux (vH^+^) and a decrease in proton conductivity (gH^+^) of the chloroplast ATP synthase (Figure 5C,D). The combination of these results suggests a significant reduction in ATP synthase activity under saline conditions, regardless of the Bc or SAP amendments.

### 2.7. Electron Transport Rate (ETR)/Photosynthetic Assimilation Rate (A_net_ Ratio)

**Figure 6:**
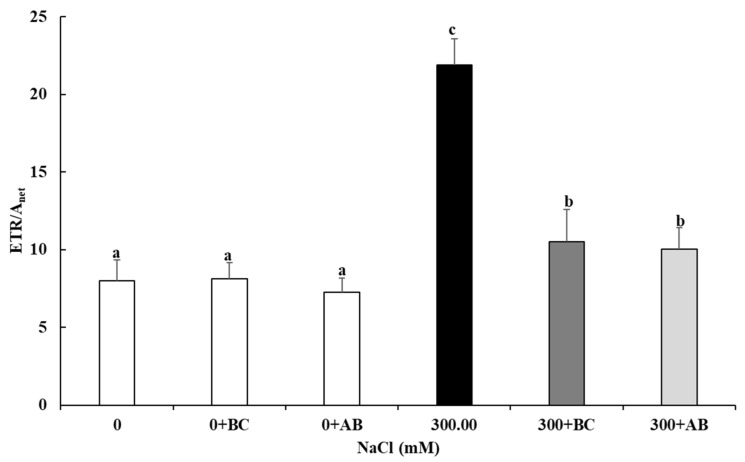
Impact of biochar (BC) or absorber (AB) amendments on ETR/A_net_ ratio of *Chenopodium quinoa* Willd. (variety ICBA-5) plants with light saturation (1200 PPFD) after culture without additional NaCl (control), or for 27 days at 300 mM NaCl. Values represent mean ± SE (n = 5), and the different letters a to c indicate significant differences between the treatments. Control (0), 300 mM NaCl (300), biochar (Bc), absorber (Ab).

The increase in the ETR/A_net_ ratio is an indicator of the risk of oxidative stress. There was no significant difference in the ETR/A_net_ ratios regardless of an amendment at 0 NaCl salinity (Figure 6). However, in those plants subjected to 300 mM NaCl salinity over 27 days, this led to nearly a tripling of the ETR/A_ne_t ratio in the treatment without any amendment. This occurred because the reduction of A_net_ (see Table 1) was much higher than the reduction of ETR (see Figure 4). In contrast to this result at 300 mM NaCl, the Bc and SAP amendments both reduced the ETR/Anet ratio by more than 50%, though, as expected, they were still higher than the 0 NaCl control. With the addition of Bc and SAP to the culture medium, the significantly reduced net ETR/A_net_ ratio also reduces the risk of oxidative stress.

### 2.8. Enzymatic Antioxidant Defense and Hydrogen Peroxide (H_2_O_2_) Content

**Figure 7:**
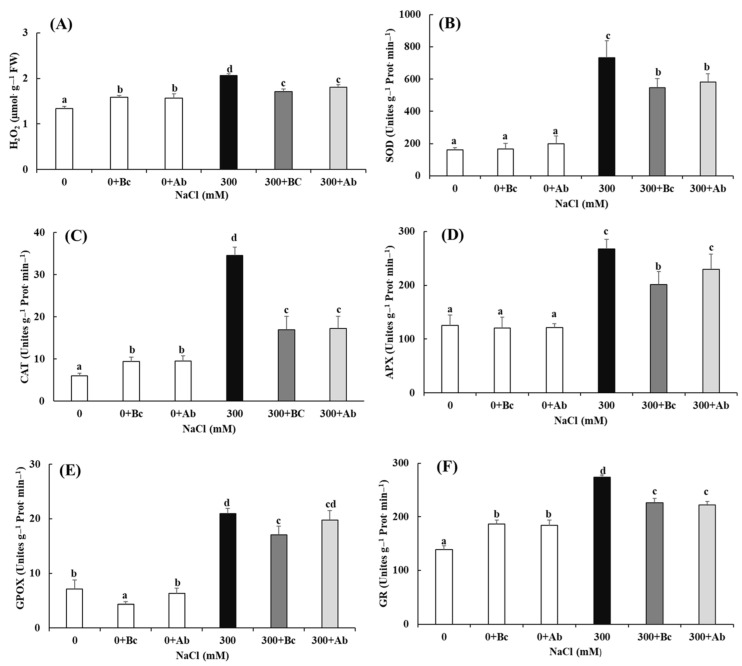
The difference in (**A**) H_2_O_2_ content and the enzymatic activities of (**B**) SOD, (**C**) CAT, (**D**) APX, (**E**) GPOX and (**F**) GR in leaves of *Chenopodium quinoa* Willd. (variety ICBA-5) plants after culture without additional NaCl or 27 days of salt treatment. Values represent mean ± SE (n = 5), and the different letters a to d indicate significant differences between the treatments. Control (0), 300 mM NaCl (300), biochar (Bc), absorber (Ab).

The Bc and SAP amendments, at 0 mM NaCl, provided a significant increase in H_2_O_2_. However, at 300 mM NaCl, these same amendments led to a significant decrease in the H_2_O_2_ content (Figure 7). There was a clear positive correlation between the H_2_O_2_ content and CAT and glutathione reductase (GR) activity in all treatments. The activities of the other measured enzymes of ROS defense SOD, APX and guaiacol peroxidase (GPOX) increased only at 300 mM NaCl (hyperosmotic salinity). 

### 2.9. Redox State of Ascorbate 

**Figure 8:**
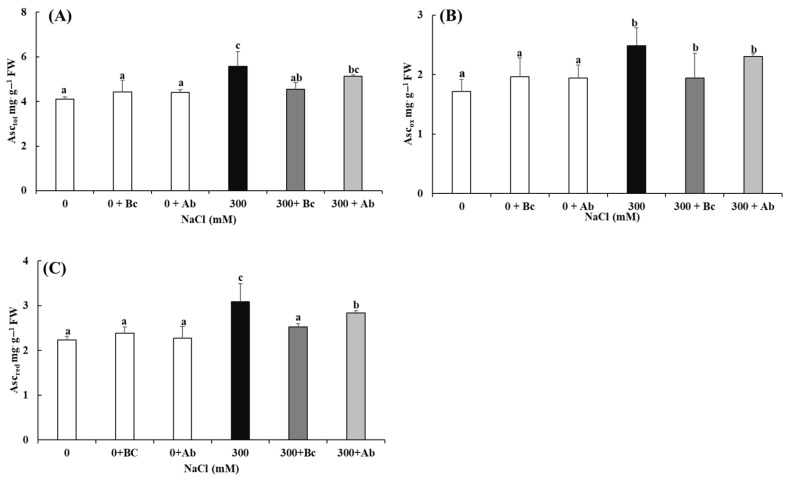
Impact of biochar or absorber amendments on the content and redox state of ascorbate in leaves of *Chenopodium quinoa* Willd. (variety ICBA-5) plants after culture without additional NaCl or 27 days of salt treatment. (**A**) Total ascorbate content, (**B**) content of oxidized and (**C**) reduced ascorbate. Values represent mean ± SE (n = 5), and the different letters a to c indicate significant differences between the treatments. Control (0), 300 mM NaCl (300), biochar (Bc), absorber (Ab).

The amendments Bc and SAP had no significant impacts on the ascorbate content (Figure 8A,C) at 0 mM NaCl salinity (0 + Bc and 0 + Ab). In agreement with the changes in the enzymatic and non-enzymatic ROS defense (Figure 7), this led to hyperosmotic salinity (300 mM NaCl), with a significant increase in the Asc_tot_, Dehydroascorbate (Asc_red_) and Ascorbate (Asc_ox_) contents (Figure 8). Moreover, the amendment of Bc and SAP at 300 mM NaCl salinity also caused a significant decrease in the total ascorbate (Asc_tot_), Asc_red_ and Asc_ox_ contents. However, there was no significant difference in the Asc_red_/Asc_ox_ ratio for all six treatments.

## 3. Discussion

Quinoa is a halophil crop characterized by its ability to resist high NaCl concentrations in soils. In this study, we tried to improve the yield production of *Chenopodium quinoa* Willd. (variety ICBA-5) in hyperosmotic salinity (300 mM NaCl) and to enhance its growth capacity with Bc or SAP amendments to the soil. It was observed in several studies on various plant species that hyperosmotic salinity reduces biomass production, mainly due to the ionic and osmotic stresses [4]. The results of the present study confirm a salt-related decrease in the leaf, stem, root and whole plant’s fresh weight (Figure 1) but also the positive effect of Bc or SAP on the fresh weight production [49]. We explain our results by the fact that Bc improved the soil moisture content through higher retention capacities. Bc, depending on the form and particle size, can decrease the soil density and increase the soil’s surface area due to its porous structure, which increases its ability to assimilate and retain water. Our study agrees that the porous structure of Bc decreases evapotranspiration and increases the soil’s aeration and water-holding capacity (WHC) [50]. In addition, it was reported that Bc could optimize the soil water content and reduce plant-available Na^+^ and Cl^-^ concentrations in soils under hyperosmotic salinity, thus maintaining a suitable soil environment for plant growth [49]. The amendment of SAP in the soil also had a positive impact on the biomass production of quinoa (Figure 1). This effect was explained by an increase in the water-holding capacity, leading to a retention of significantly more water in the rhizosphere of the plant and, consequently, a reduction in oxidative stress [51,52]. Additionally, it was shown that the introduction of Bc into the soil can help to improve plant resilience when grown in saline sites by enabling a higher root surface area and reduced drainage of water [53].

### 3.1. Chlorophyll Content and CO_2_/H_2_O Gas Exchange (Light-Independent)

Components participating in photosynthetic mechanisms, such as photosynthetic pigments, photosystems, electron transport systems, gas-exchange processes and enzymes involved in carbon metabolism, are important for photosynthetic efficiency and could be potentially affected by abiotic stresses such as salinity [54]. 

The destruction and disruption of the active photosynthetic mechanism in the leaves, which can thus cause chlorosis and early leaf senescence, is one of the first and most obvious adverse effects of hyperosmotic salinity on the plant [19] and could also be shown in this study for quinoa plants at 300 mM NaCl (Table 1). The decline in the chlorophyll content at 300 mM NaCl was explained by an increase in chlorophyllase enzyme activity [55] and with a limited nitrogen uptake [56]. The increase in the chlorophyll content in leaves of quinoa plants grown with Bc can be explained by the increased accessibility of nitrogen in the soil and, consequently, higher nitrogen availability and higher chlorophyll content in leaves [57,58,59,60,61,62]. Additionally, it was reported that the saline application significantly reduced total leaf chlorophyll and also the carotenoid content in beans, and that the Bc amendments applied at 5 t ha^−1^ and 15 t ha^−1^ to the topsoil mitigated these negative effects [63]. In agreement with the previously published studies, the Bc-induced increase in the chlorophyll content in quinoa leaves was accompanied by an increase in CO_2_ fixation (A_net_: from 6.36 to 12.51 CO_2_ µmol m^−2^ s^−1^) [64,65,66,67]. The additional increase in transpiration (Tr) and stomatal conductance (g) (see Table 1) can be attributed to the Bc-induced increase in the water-holding capacity of the soil, which again can be related to the porous structure of the Bc in the soil [68,69,70]. We conclude, on the basis of several similar studies, that the Bc amendment can enhance A_net_ by the improvement in the soil’s water-holding capacity and increase in the availability of N or P in the soil, which can lead to the amelioration of the plant yield and therefore the enhancement of the plant’s fresh weight [71,72,73]. The same positive effect was observed with the amendment of the SAP (300 + Ab). SAP clearly supported the increased leaf chlorophyll content, stomatal conductance and leaf transpiration, which positively affected the plants’ CO_2_ fixation. Our findings are in line with other studies [74,75], which reported that SAP significantly increased the chlorophyll concentration in corn under drought stress. In addition, it was reported that the SAP amendment increased the water-holding capacity and ion-exchange capacity of the soil [76]. The authors concluded that both soil ameliorations (SAP and Bc) helped to avoid salinity-induced damage to the photosynthetic apparatus. 

### 3.2. Light-Dependent Reaction: Quantum Yields and Energy Conversion in PSII

Chlorophyll molecules capture light energy and use it in photochemical reactions (YI and YII) to drive photosynthesis and, finally, to transfer the energy to ADP and NADP^+^, of which the latter is on the acceptor side of PSI. Salinity can affect the function of thylakoids in chloroplasts and their photosynthetic performance. In our study with quinoa, the presence of NaCl in the soil led to reduced yields (YII) and reduced electron transfer rates (ETR) in photosystem II (Figure 4). This impact correlates with the decreased chlorophyll content and gas exchange (Figure 3 and Table 1). It has been reported by several authors that high NaCl soil concentrations can lead to a decrease in photosynthetic efficiency (PSI and PSII, [77] and to an enhancement of photoinhibition [78]). However, reduced photosynthetic efficiency and ETR are not generally a sign of a destructive effect. It can even be essential when the demand for energy for the non-light-dependent reaction is low, as shown for *Chenopodium quinoa* in this study (see Figure 4). As expected, hyperosmotic salinity in quinoa led to a decrease in the net-CO_2_ fixation (Table 1) similar to ETR (Figure 4), indicating a salinity-induced higher risk of oxidative stress. The disproportionate supply of electrons (e) to the NADP reaction center can cause a significant accumulation of electrons at the end of the electron transport chain and favor ROS formation instead of CO_2_ reduction [79]. The addition of Bc and SAP to the culture medium significantly reduced the ETR/A_net_ ratio and consequently led to a lower risk of oxidative stress. The low ETR/A_net_ ratios of plants grown in soil with Bc or SAP also showed a negative correlation to the associated growth response (Figure 6), indicating a higher availability of energy for biosynthesis instead of ROS defense (Figure 7 and Figure 8).

While light is essential for photosynthesis, it can also lead to light-induced damage when the absorbed light energy exceeds the capacity of the photosynthetic machinery. To avoid this, the excess photons and electrons need to be dissipated. This occurs through photoinhibition or a rapidly inducible non-photochemical quenching process Y(NPQ) in which the absorbed excess light energy is dissipated as heat [80]. Our results showed that salinity led to an increase of Y(NPQ) and Y(NO) in quinoa leaves and are in accordance with those that were found by [81], who reported the same result for cucumber. According to [82], Y(II), Y(NPQ), and Y(NO) are in ‘competition’, so an increase in one results in a decrease in the two others. Under stressful conditions, high Y(NO) values and low Y(NPQ) or Y(NPQ)/Y(NO) values reflect an inefficient ability for photoprotective reactions, which will eventually lead to photodamage [83]. However, this was not the case in quinoa. In accordance with the results of [84], quinoa released most of the light energy in the form of Y(II) and Y(NPQ), and the latter compensated for the decrease in the first one at a high salinity. It was shown that the Y(NPQ) value increased in tolerant varieties and decreased in sensitive varieties under hyperosmotic salinity [85]. Under the control or salt conditions, the fraction of energy dissipated as heat via regulated non-photochemical quenching (Y NPQ) or non-regulated non-photochemical energy loss (Y(NO)), and was not significantly affected by the Bc or SAP amendment. Moreover, there was the expected close correlation between the salt-induced increase of Y(NPQ) and the increase in the leaf temperature differential.

### 3.3. Proton Motive Force

The electrochromic shift (ECS) signal reflects changes in the electric field across the thylakoid membrane that, in turn, reflects the build-up of the thylakoid proton motive force by photochemistry and its subsequent utilization by ATP synthesis [86]. The ECS decay, during brief dark intervals, can provide information about the light-driven fluxes of electrons and protons, the extent of energy storage in the thylakoid proton motive force, the activity of the chloroplast ATP synthase, and together with the ETR, the activation of the cyclic electron flow. These responses are sensitive to environmental conditions and have an impact on the CO_2_/H_2_O gas exchange, such as hyperosmotic salinity [87]. Previous studies indicated a close relationship between the values of the ECS parameters and the efficient regulation of electron transport and photoprotection in hypersaline conditions [88]. The maximum amplitude of the signal (ECS_tau_), as a measure of the proton motive force, significantly decreased under hyperosmotic salinity in quinoa, which is in agreement with similar treatments with wheat varieties [89]. The product of the proton motive force (parameter ECS_tau_) and the thylakoid conductivity to protons (parameter gH^+^) can serve as an estimate of the proton flux (ECS_tau_·gH^+^) [90]. The decrease in ECS_tau_ was partially buffered (by ca. 50%) in quinoa by an increase in the proton conductivity (gH^+^), leading to an overall decrease in the steady-state rate of the proton flux (vH^+^) (Figure 5) through the chloroplast ATP synthase. The estimated proton flux (ECS_tau_·gH+) correlated nicely with the ETR (see Figure 4). Even the impact of hyperosmotic salinity showed a close correlation by a ≈50% reduction in both parameters. Therefore, we do not assume that the shown increase in proton conductivity can be explained by leaks of H^+^ through the thylakoid membrane, as discussed by [87], but a coordinated interaction between photosystem 2 (PSII) and photosystem 1 (PSI). This hypothesis is confirmed by the values of MDA and proline, both indicators of oxidative stress (Figure 3). Both the Bc and SAP amendments led to a significant reduction in MDA and proline in hyperosmotic salinity levels, but had hardly any effect on ECS_tau_, gH^+^ and vH^+^.

The combination of these results proves the assumption of a significant reduction in ATP synthase activity under saline conditions. In environmental stresses such as salinity, when assimilation is limited by low CO_2_ availability, the activity of the ATP synthase is rapidly and reversibly decreased, slowing the efflux of protons from the thylakoid lumen [91], initiating the downregulation of the light reactions that involve the activation of the photoprotective qE (or Y(NPQ)) response and the slowing of electron transfer at the cytochrome b6f complex [92,93].

### 3.4. Indicator of Oxidative Stress

Proline is one of the most common osmolytes produced by plants under hyperosmotic salinity. This low-molecular-weight osmolyte helps plants to resist osmotic stress [94]. Osmolytes such as proline play an essential role in osmotic adjustment and also in guard cells by scavenging ROS [95]. Proline can generate toxic and harmful effects on the plant when it is accumulated in higher concentrations inside the plant cells. Among the symptoms that are caused by proline are alterations that affect the ultrastructure of chloroplasts and mitochondria, in addition to several aspects of programmed cell death [96,97]. According to [98], proline causes, at high concentrations, the generation of reactive oxygen species (ROS) through the intervention of NADPH oxidases, leading to the appearance of toxic symptoms. The toxic effect of proline can be attributed to the fact that a high concentration of proline activates the P5C/proline cycle [99]. Hyperactivation of this cycle induces increased electron genesis from the incomplete oxidation of proline that can exceed the transfer potentials of the mitochondrial chain, resulting in increased electron transfer to O_2_ and leading to the formation of ROS [100]. The results of our study revealed that the salinity-induced high increase in the proline content in quinoa leaves (Figure 3; var ICBA-5) and the related possible toxic or harmful effects could be reduced significantly by both the Bc and SAP amendments. 

Moreover, the increase in MDA and hydrogen peroxide (H_2_O_2_) contents, triggered by 300 mM NaCl treatment, was significantly reduced by the Bc and SAP amendments. MDA (malondialdehyde) accumulation is known as an index of oxidative damage and increased lipid peroxidation caused by ROS [101]. Hydrogen peroxide (H_2_O_2_) is one of these reactive oxygen species (ROS) and is usually produced in large quantities by plants in response to various stressful conditions. It can aggressively damage cellular membranes and organic molecules [28]. We assume that the Bc and SAP amendments mitigated oxidative stress levels in salinity-treated plants by inhibiting H_2_O_2_ production in comparison to plants without amendments. This assumption is confirmed by studies where the content of MDA and H_2_O_2_ remarkably increased in beans via saline irrigation, while these increases were blocked significantly by the addition of Bc to the soil [102]. The authors assumed that amendments such as Bc (and SAP) could stimulate or relieve the antioxidant system, enabling the control of ROS levels in plant tissue [103].

Plants use various means of protection and survival to withstand difficult circumstances, such as the activation of a signaling pathway, the use of a quality control system to survive under the effect of unfavorable factors and the production of antioxidant enzymes in different quantities. According to our study (Figure 3 and Figure 7), the addition of Bc to soil reduced the activity of antioxidant enzymes [104], including CAT, APX, SOD, GPOX and GR, under saline conditions. It also degraded ROS, proline and H_2_O_2_ and inhibited lipid peroxidation in plant cells (MDA). The application of Bc in the culture medium under saline conditions led to a reduction in the MDA, proline and H_2_O_2_ concentrations, leading to an increased supply of energy for the biomass production of leaves, stems and roots and, thus, an increase in the fresh weight of the whole quinoa plant under salt stress conditions (Figure 1). Our results are in agreement with studies that reported that Bc could reduce the impacts of salinity stress [44]. The increase in ROS, synthesized by plants, is often mainly due to increased salinity in the soil. The decrease in the proline, MDA and H_2_O_2_ concentrations in plants treated with 300 + Ab can also be explained by the ability of SAP to conserve and store water. It was shown that the application of SAP increased the soil’s capacity for water retention, preventing water deficiency for enhanced growth [105]. Thus, under high concentrations of hyperosmotic salinity, the amendment of SAP would ensure more plant-available water in the soil and reduce oxidative stress at the phyto-physiological levels, resulting in better growth and biomass production.

### 3.5. Enzymatic and Non-Enzymatic Antioxidants

Antioxidant enzymes represent a major ROS-scavenging force and are of eminent importance for stress resistance in plants and controls, together with non-enzymatic antioxidant defense and the regulation of ROS levels through strict compartmentalization [106,107] by a series of redox reactions for ROS elimination. The significance of antioxidant enzymes has been documented by many studies reporting the positive correlation between the expression of these enzymes and plant stress resistance [108]. The deregulation of the antioxidant machinery may lead to the excessive accumulation of ROS in plants, with negative consequences in terms of plant performance and development. Quinoa responded in hyperosmotic salinity to oxidative stress by the activation of plastidic, cytosolic, mitochondrial and peroxisomal SODs, which decompose O_2_^●−^ to H_2_O_2_ [30]. H_2_O_2_ is the most stable of the so-called reactive oxygen species (ROS) and an unavoidable by-product of photosynthesis, respiration and photorespiration [109,110,111]. H_2_O_2_ plays a crucial role as a signaling molecule and regulates plant growth, development, acclimatory and defense responses [112]. H_2_O_2_ positively modulates cell production and root elongation under well-watered conditions in fully-sized plants [113] and may explain the increase in root growth and H_2_O_2_ content in the Bc- and SAP-amended 0 NaCl treatments of this study (Figure 1 and Figure 7). Because of the consumption of reduced power and energy, this apparently wasteful process of H_2_O_2_ generation could act up to a distinct degree as an electronic valve and ease the electronic burden of the photosynthetic machinery [112]. However, it was observed that ROS, particularly H_2_O_2_, increased specifically in the apical region of the growth zone under water stress and caused downregulation of cell production and root growth inhibition [114]. These results are in agreement with our findings and show that H_2_O_2_ levels regulate cell production and root elongation in both well-watered and water-stressed conditions. 

Consequently, CAT was activated by quinoa (Figure 7) in the hyperosmotic salinity treatments to catalyze the decomposition of H_2_O_2_ to H_2_O and O_2_. CAT isozymes are localized in peroxisomes [115] and play important roles under unfavorable conditions for plants. Redox-related processes are strictly regulated by such proteins as thio- and glutaredoxins, which can undergo reversible oxidation/reduction and can be activated/inactivated in response to the cellular redox state [116]. Quinoa also tried to maintain balance in cellular H_2_O_2_ by activation of enzymes of the ascorbate–glutathione cycle, such as APX and GR. APXs, as heme-containing peroxidases, detoxify H_2_O_2_ via the electron transfer from ascorbate to form monodehydroascorbate (MDHA) and H_2_O (Figure 7 and Figure 8). Furthermore, the aim to recover the pool of reduced glutathione consumed by GPOX and DHAR activity by an increase in GR in a NADPH-dependent reaction was clearly detectable for quinoa in hyperosmotic salinity. This was necessary because quinoa also activated glutathione peroxidases (GPxs, Figure 7), a non-haem-thiol peroxidase that catalyzes the reduction of hydrogen peroxide (H_2_O_2_) to water and other lipid hydro-peroxides by reduced glutathione (GSH) [117]. 

The cellular antioxidant capacity is tightly coupled with the maintenance of redox homeostasis by redox buffers such as ascorbate [107]. In our study, we could show that quinoa responds to oxidative stress by enhanced synthesis of ascorbate. Quinoa increased the contents of total, reduced and oxidized ascorbate significantly in the hypersaline conditions (Figure 8). Ascorbate can directly decompose ROS and is essential for preserving the ROS content at physiological levels. The high reduction state is reported by several authors and has been related to enhanced plant resistance to harmful conditions and increased antioxidant capacity [107]. However, the antioxidant capacity was still not sufficient in hyperosmotic salinity to avoid oxidative stress, as shown by the ETR/A_net_ ratio, MDA, proline and H_2_O_2_. 

This study showed that the amendment of Bc and SAP significantly reduced the oxidative stress and the enzymatic and non-enzymatic ROS responses of quinoa in hyperosmotic salinity. This may be explained, at least partially, by the significantly reduced H_2_O_2_ expression. The presented results are in line with the studies on the olive tree (*Olea europaea*) [118], *Oryza sativa* [119], *Glycine max* [120], *Beta vulgaris* [121] and *Zea mays* [122]. We can deduce that the amendments of Bc and SAP have an important role in the resistance of hyperosmotic salinity and, subsequently, the diminution of antioxidant activity. Both amendments mitigate hyperosmotic salinity, CAT, SOD, GR and GPOX activity, the ETR/A_net_ ratio, and proline, H_2_O_2_ and MDA contents in the leaves of quinoa. We conclude that SAP or an organic amendment like Bc can reduce the negative impacts of hyperosmotic salinity on ROS development and consequently enable plants to reduce their anti-oxidative responses. In fact, ref. [123] showed that Bc declined ascorbate peroxidase (APX) and glutathione reductase (GR) activities of *Zea mays* under salinity treatment. Similarly, ref. [102] showed that Bc treatments lowered antioxidant enzyme activities and oxidative stress in salt-stressed bean plants. They also suggested that the beneficial effects of Bc can be due to decreased MDA and H_2_O_2_ development. Furthermore, the Bc amendment decreased the contents of ABA under salinity stress conditions. Ref. [124] reported that Bc alleviated the negative effects of salt stress on bean seedlings by reducing the Na concentration and ABA content. Therefore, it was postulated that Bc can generally mitigate the negative effects of salinity on plants.

## 4. Materials and Methods

### 4.1. Plant Material and Growth Conditions

The experiment was conducted in a greenhouse at the Institute of Plants Ecology, Giessen, Germany, under controlled conditions at a temperature of 24 °C/15 °C (day/night), relative humidity of 55–60% and a photoperiod of 16/8h (day/night). *Chenopodium quinoa* seeds of the variety ICBA-5 were provided by the Seedbank International Center for Biosaline Agriculture (ICBA, Dubai, UAE) and collected in the Center of Biotechnology of Borj_Cedria (CBBC, Hamman-Lif, Tunisia). Seeds were sterilized and germinated in pots filled with a mixture of black soil. Three weeks after their germination, the plants were transferred into pots of 1.5 kg containing soil (70% mixture of soil and 30% sand) mixed with 11.25 g of perlite to favor a better aeration. The mixtures were prepared per batch before being distributed among the pots. Biochar and superabsorbent polymers (SAP, Stockosorb, Agrinova GmbH, Quirnheim, Germany) were used as amendments for soil improvement. Coniferous wood and hardwood chips (1:4 ratio by weight) were mixed to produce biochar through pyrolysis in a 36 h cycle at 750 °C using a Schottdorf-type reactor (Carbon Terra, Augsburg, Germany). Both amendments, SAP (3 g SAP per pot and 1.5 kg soil ≙ 0.2 g/100 g) and Bc (45 g per pot and 1.5 kg soil ≙ 3 g/100 g) were distributed among the individual pots and mixed thoroughly with the soil to reach homogeneous soil conditions within one approach. Different concentrations of SAP and BC were selected to achieve the same water-holding capacity in both approaches.

The salt treatment started on day 25. NaCl was added to the soil of half of the cultured plants stepwise (50 mM d^−1^) until the required concentration of 300 mM NaCl was reached and maintained for a further 27 days. The pots were divided into 6 groups, with 4 pots for each group: control (0) or salinity (300 mmol NaCl (300)) without any amendment; control (0 + Bc) or salt (300 + Bc) with the Bc addition; and control (0 + SAP) or salinity (300 + SAP) with a superabsorbent addition. 

The plants were irrigated with diluted (half-strength) nutrient solution (modified after Hewitt, 1966) containing 3.5 mM Ca(NO_3_)_2_, 3.0 mM KNO_3_, 1.5 mM MgSO_4_, 1.6 mM KH_2_PO_4_, 0.6 mM K_2_HPO_4_, 3 μM Fe–K-EDTA, 0.05 μM H_3_BO_3_, 0.5 μM MnSO_4_, 0.04 μM CuSO_4_, 0.05 μM ZnSO_4_ and 0.02 μM (NH_4_)_6_Mo_7_O_24_). Care was taken to ensure that all six treatments received the same quantity of nutrients until the final harvest on day 52. 

### 4.2. Plant Growth Determinations

During the harvest, the water content (in %), EC (dS/m) and temperature (T) were measured in the soil with a WET 2 sensor in combination with a HH2 moisture meter (Delta-T Devices (UK)). Subsequently, the roots were separated from the soil before they were washed carefully with water and dried superficially with paper towels. Afterward, the plants were fixed at the root base to a tripod to photograph them. Finally, the total plant weight, root, stem and leaf fresh weight were taken from each sample before drying at 80 °C for 72 h in the oven to determine the dry weight. 

#### 4.2.1. Chlorophyll Content (Total Chlorophyll)

The chlorophyll content was measured using the SPAD-502 (Konica Minolta, Langenhagen, Germany), and the measurements (n > 4 replicates) were taken just before the harvesting of plants in the morning on the first fully developed leaf (the third or the fourth nodes of all plants). We chose to measure chlorophyll with the SPAD because it is a nondestructive method that enables us to follow the development of the chlorophyll content during growth and to compare the impact of salinity with other methods bound to the surface area, such as the CO_2_/H_2_O gas-exchange and chlorophyll fluorescence.

#### 4.2.2. Chlorophyll Fluorescence and Absorbance-Based Parameters (Light-Dependent Reactions of Photosynthesis)

According to the methods described previously [86], the photosynthetic activity of the light-dependent reaction was determined at the third and the fourth nodes of plants (4 replicates, n = 4) at the first fully developed leaf. Light saturation curves were measured in the time between 10 a.m. to 3 p.m. with a MultispeQ V 2.0 (PHOTOSYNQ INC. 325 E. Grand River Ave. Suite #331, East Lansing, MI, USA) using the protocol “Photosynthesis RIDES Actinic Series 10 2x2000”, programmed by Grueters in 2022. We measured the fluorescence base parameters of plant leaves, including the electron transport rate (ETR), the quantum yield of photosystem II (Y(II)), non-photochemical quenching (Y(NPQ)) and the fraction of energy that is passively dissipated in the form of heat and fluorescence (Y(NO)). We also studied the photosystem II redox state to derive the ATPase activity with absorbance-based parameters like steady-state proton flux (vH^+^), proton conductivity (gH^+^) and the magnitude of electrochromic shift (ECS_tau_) [93]. 

#### 4.2.3. CO_2_/H_2_O Gas Exchange (Light-Independent Reactions of Photosynthesis)

Measurements were taken at the youngest fully developed leaf (the third and the fourth nodes of four replicate plants). The following CO_2_/H_2_O gas-exchange parameters were measured with a LI-6400XT portable photosynthesis system (LI-COR, Inc., Lincoln, NE, USA) during the day between 10 a.m. and 3 p.m. at light saturation (1200 PPFD) and a net atmospheric CO_2_ concentration of 400 ppm with the automatized standard settings of the LI-COR standard software v6.3.4.: Stomatal conductance (g_s_ in mmol H_2_O m^–2^ s^–1^), net CO_2_ assimilation rate (A_net_ in μmol CO_2_ m^–2^ s^–1^), intercellular CO_2_ concentration (C_i_ in ppm CO_2_), transpiration rate (Tr in mmol H_2_O m^–2^ s^–1^) and an instantaneous plant water-use efficiency (PWUE, µmol CO_2_ mmol H_2_O^−1^). 

#### 4.2.4. Proline Measurements

The proline concentration was determined according to the method of [125]. For the extraction, 200 mg of leaf fresh weight (n = 4) was homogenized in 4 mL of sulphosalicylic acid (3% *w/v*) and then mixed with an acid ninhydrin solution (2 mL) and glacial acetic acid (2 mL). The mixture was heated in a water bath (at 90 °C for 1 h), and the reaction was stopped in an ice bath. Subsequently, 4 mL of toluene was added into each tube to extract the proline before the absorbance of the toluene fraction (aspired from the liquid phase) was measured at λ 520 nm with the UV/VIS spectrophotometer CAMSPEC M550 double beam (Spectronic CamSpec, Leeds, UK). The proline concentration was determined as µmol proline g^−1^ FW using a standard curve of different concentrations of proline. 

#### 4.2.5. MDA Measurements

Lipid peroxidation was measured according to the method of [126]. A total of 50 mg of fresh leaf material (n = 4) was homogenized with a prechilled mortar and pestle in 2 mL of ice-cold trichloroacetic acid TCA (1%, *w*/*v*) and centrifuged at 10,000 rpm for 10 min at 4 °C. A total of 2 mL of supernatant was mixed with 0.5 % (*w*/*v*) of thiobarbituric acid (TBA) and heated at 95 °C for 30 min before being rapidly cooled in an ice bath. Subsequently, the samples were centrifuged (10,000 rpm for 10 min at 4 °C), and the supernatant absorbance was measured at λ 532 and λ 600 nm with the UV/VIS spectrophotometer CAMSPEC M550 double beam (Spectronic CamSpec, Leeds, UK). The concentration of MDA was calculated with the help of the extinction coefficient 155 mM^−1^ cm^−1^.

#### 4.2.6. Hydrogen Peroxide (H_2_O_2_) Content

The hydrogen peroxide (H_2_O_2_) concentration was measured according to the method previously described by [127]. A total of 0.5 g of fresh leaf samples (n = 4) were ground and mixed in 5 mL of 1% (*w*/*v*) ice-cold trichloroacetic acid (TCA) and centrifuged at 14,000× *g* for 20 min at 4 °C. A total of 0.5 mL of supernatant was mixed with 0.5 mL of potassium phosphate buffer (10 mM, pH 7.0) and 1.5 mL of potassium iodide (1 M) at a ratio of 2:1 (*v*/*v*). The absorbance was measured at λ 390 nm with the UV/VIS spectrophotometer CAMSPEC M550 double beam (Spectronic CamSpec, Leeds, UK). The H_2_O_2_ content was calculated using a standard curve of different concentrations of H_2_O_2_. 

### 4.3. Assay to Determine Protein Content and Antioxidant Enzyme Activity 

A total amount of 100 mg fresh leaves (n = 4 replicates) was homogenized with ice-cold sodium phosphate buffer (50 mM, pH 7.2) mixed with 1 mM ascorbic acid, 1 mM dithiothreitol (DTT), 0.1% of triton, 10 mM ethylene diamine tetra acetic acid (EDTA, disodium salt) and 10% (*w*/*v*) Polyvinylpolypyrrolidone (PVPP). The homogenate was centrifuged at 12,000× *g* for 20 min at 4 °C. The supernatant was recovered and stored in a small Eppendorf at −80 °C [128]. 

Leaf protein concentrations were determined after mixing the supernatant with an acid solution of Coomassie–Brillant–Blau G-250 and subsequent incubation in the dark for 10 min, according to [129]. Using a UV/VIS spectrophotometer CAMSPEC M550 double beam (Spectronic CamSpec, Leeds, UK), the absorbance was measured at λ 595 nm. Soluble protein concentrations in the enzyme extracts were estimated using a standard curve of different concentrations of bovine serum albumin (BSA). The same spectrophotometer was used to measure the following enzymatic activities:

CAT Activity

The measurement of CAT activity was conducted according to the method of [130]. The reagent mixture (3 mL) was assayed by mixing 100 µL of the enzyme extract, 50 mM of phosphate potassium buffer (pH = 7) and 50 mM of hydrogen peroxide (35%). The catalase activity of fresh leaves was estimated by monitoring the decrease in the absorbance at 25 °C and at a wavelength of λ 240 nm.

Ascorbate Peroxidase (APX)

The ascorbate peroxidase (APX) activity was measured using the method of [131]. The reaction mixture (3 mL) consisted of a mixture of 50 mM potassium phosphate buffer (pH = 7.0), 2 mM H_2_O_2_, 0.5 mM of Ascorbate, 0.2 µM EDTA, 0.5 mM of ascorbate, 2 mM H_2_O_2_ and 50 µL of enzyme extract. The reaction was initiated by the addition of H_2_O_2_. Ascorbate peroxidase was assayed by monitoring the decrease in absorbance at λ 290 nm. The molar extinction coefficient was 2.8 mM^−1^ cm^−1^.

Guaiacol Peroxidase

The GPX activity was estimated according to [132] by recording the increase in absorbance at λ 470 nm due to a tetra-guaiacol formation (ɛ = 26.6 L mol^−1^ cm^−1^). The reaction mixture (3 mL) contained 50 mM of potassium phosphate buffer (pH 7.0), 2 mM H_2_O_2_, 2.7 mM Guaiacol and 50 µL of enzyme extract. The enzyme activity was calculated as the percentage of inhibition per min. The molar extinction coefficient was 26.6 L mol^−1^cm^−1^.

Glutathione Reductase

The activity of glutathione reductase (GR, EC 1.6.4.2) [133] was detected as oxidation of β-NADPH at λ 340 nm (ɛ = 6.2 L mol^−1^ cm^−1^). The reaction mixture (3 mL) contained 100 mM Tris-HCl (pH 7.8), 0.5 mM GSSG, 0.03 mM_β-NADPH, 5 mM EDTA and 100 µL of enzyme extract. The molar extinction coefficient was 6.2 L mol^−1^ cm^−1^. 

#### Superoxide Dismutase 

The superoxide dismutase activity could be determined because of its ability to prevent the photochemical reduction of nitroblue tetrazolium chloride (NBT) at λ 560 nm. According to [134], we prepared a reagent based on 10 mM L-methionine, 0.1 mM of nitroblue tetrazolium chloride (NBT) and 0.75% Triton X-100 in 50 mM potassium phosphate with a pH of 7.8 and stored it in a dark bottle. A total of 1 µL of this reagent was added to the reagent medium (3 mL) containing 40 µL of the enzyme extract, followed by 10 µL of 0.12 mM riboflavin. The mixture was prepared twice; one of them was incubated under fluorescent lamps (40 W) for 7 min, and the second one was kept in the dark to be used as a blank for measurements. The absorbance of the mixture was detected at λ 560 nm. The enzyme activity was calculated as the percentage of inhibition per minute.

### 4.4. Extraction and Determination of Non-Enzymatic Antioxidant Ascorbate (AsA) and Dehydro-Ascorbate (DHAsA)

Based on the method described by [135], frozen leaf samples (0.4 g, n = 4) were ground in liquid nitrogen and homogenized in 2 mL of ice-cold 6% TCA. The mixture was centrifuged at 15,000× *g* for 20 min at 4 °C. Ascorbate (AsA) and dehydro-ascorbate (DHAsA) were determined in the supernatant with a dipyridyl assay based on the reduction of Fe^3+^ by reduced ascorbate, followed by the complex formation between Fe^2+^ and bipyridyl, which absorbs at λ 525 nm. Total ascorbate was determined after the reduction of DHAsA to AsA by reacting it with dithiothreitol. A standard curve was prepared for the estimation of total ascorbate (with pretreatment DTT) and DHAsA (subtracting AsA from the total ascorbate). 

### 4.5. Statistics

Statistical analyses were carried out by a two-way analysis of variances using Sigma Plot software 11.0. For this purpose, four replicates were used for the data analysis. A two-way analysis of variance (ANOVA) was performed to test the independence of variation among conditions (equal variance test) and the normal distribution of data of each variable (Shapiro–Wilk). The Holm–Sidak method (all pairwise multiple comparison procedures) was used to check whether the means of the posterior homogeneous subgroups differed significantly at *p* < 0.05. We also used Sigma Plot software 11.0 to determine the function of the light-response curves (R^2^ > 0.98). The mathematical description of the light-response curve is given by the non-linear exponential function f(x) = a − exp[b(−x)]c [136].

## 5. Conclusions

The results presented in this study support our hypothesis that the application of the Bc and/or SAP amendments to the soil increases the salinity resistance of *Chenopodium quinoa.* These amendments both increase the plant’s available water, which improves the plant’s water relations and thereby helps to stabilize photosynthesis and buffer the development of reactive oxygen species with photoprotective mechanisms (non-enzymatic and enzymatic defenses against ROS by enzymes of the Halliwell-Asada pathway). This study confirms that soil amendments can change the dynamics of the soil–plant–atmosphere (SPAC) continuum and can help to improve plant performance in saline soils. In the face of climate change-induced salinity stress, we recommend that researchers intensify their efforts to improve soil conditions by using a wider range of soil amendments in combination with plant growth-promoting microorganisms. Existing and new soil amendments (ideally bio-derived rather than from petroleum), when made in combination with resilient genetic variants resulting from crop breeding, are needed to open, maintain and even expand the land areas suitable for agriculture to support a rapidly expanding global population in a time of climate change. 

## Figures and Tables

**Table 1:** Effects of biochar or absorber amendments on leaf gas exchange of *Chenopodium quinoa* Willd. (ICBA-5 variety) with light saturation (1200 PPFD) after culture in nutrient solution or with 27 days of salt treatment. Net CO_2_ assimilation (A_net_), transpiration rate (Tr), substomatal CO_2_ concentration (Ci), stomatal conductance (gs) and water-use efficiency (PWUE = A_net_/Tr) values represent mean ± SE (n = 5), and the different letters a to d indicate significant differences between the treatments (Tukey test, *p* < 0.05). Control (0), (300 mM NaCl), biochar (Bc), absorber (Ab).

NaCl (mM)	A_sat_ (µmol m^−2^ s^−1^)	Tr (mmol m^−2^ s^−1^)	Ci	g_s_ (mmol H_2_O m^−2^ s^−1^)	PWUE (µmol CO_2_ mmol H_2_O^−1^)
0	21.50 ± 1.79 c	2.9 ± 0.33 c	291.67 ± 17.31 d	0.22 ± 0.06 c	7.50 ± 1.38 b
0 + Bc	22.84 ± 1.96 c	2.88 ± 0.45 c	186.19 ± 12.00 c	0.18 ± 0.03 c	8.13 ± 1.98 b
0 + Ab	26.95 ± 2.42 d	6.79 ± 0.42 d	304.44 ± 14.87 d	0.66 ± 0.09 d	3.97 ± 0.41 a
300	6.36 ± 0.30 a	1.03 ± 0.01 a	113.89 ± 25.45 a	0.06 ± 0.01 a	6.16 ± 0.35 b
300 + Bc	12.51 ± 0.58 b	1.74 ± 0.30 b	224.14 ± 24.50 b	0.14 ± 0.02 b	7.37 ± 1.66 b
300 + Ab	13.66 ± 1.30 b	1.58 ± 0.34 b	128.91 ± 21.84 a	0.12 ± 0.03 b	8.93 ± 2.25 b

## Data Availability

Data is contained within the article.

## Abbreviations

Biochar (Bc), superabsorber polymers (SAP), fresh weight (FW), water-use efficiency (PWUE), net CO_2_ assimilation rate (Anet), stomatal conductance (gs), electron transport rate (ETR), quantum yield of regulated non-photochemical energy loss in PS II (Y(NPQ)), electron (e), photosystem 2 (PSII), steady-state rate of proton flux (gH^+^), proton conductivity (vH^+^), electrochromic shift (ECS_tau_), reactive oxygen species (ROS), dehydroascorbate (oxidized form) (DHAsA), ascorbate (reduced form) (AsA), hydrogen peroxide (H_2_O_2_), malondialdehyde (MDA), catalase (CAT), ascorbate peroxidase (APX), glutathione reductase (GR), superoxide dismutase (SOD), guaiacol peroxidase (GPOX).
